# Theory-Informed Interventions to Improve the Quality of Tuberculosis Evaluation at Ugandan Health Centers: A Quasi-Experimental Study

**DOI:** 10.1371/journal.pone.0132573

**Published:** 2015-07-14

**Authors:** Lelia H. Chaisson, Achilles Katamba, Priscilla Haguma, Emmanuel Ochom, Irene Ayakaka, Frank Mugabe, Cecily Miller, Eric Vittinghoff, J. Lucian Davis, Margaret A. Handley, Adithya Cattamanchi

**Affiliations:** 1 Division of Pulmonary and Critical Care Medicine, San Francisco General Hospital, University of California San Francisco, San Francisco, California, United States of America; 2 School of Medicine, Makerere University College of Health Sciences, Kampala, Uganda; 3 Infectious Diseases Research Collaboration, Kampala, Uganda; 4 National Tuberculosis and Leprosy Control Programme, Uganda Ministry of Health, Kampala, Uganda; 5 Department of Epidemiology and Biostatistics, University of California San Francisco, San Francisco, California, United States of America; 6 Curry International Tuberculosis Center, San Francisco General Hospital, University of California San Francisco, San Francisco, California, United States of America; 7 University of California San Francisco Center for Vulnerable Populations, San Francisco General Hospital and Trauma Center, San Francisco, California, United States of America; The Foundation for Medical Research, INDIA

## Abstract

**Background:**

Tuberculosis (TB) remains under-diagnosed in many countries, in part due to poor evaluation practices at health facilities. Theory-informed strategies are needed to improve implementation of TB evaluation guidelines. We aimed to evaluate the impact of performance feedback and same-day smear microscopy on the quality of TB evaluation at 6 health centers in rural Uganda.

**Methods:**

We tested components of a multi-faceted intervention to improve adherence to the *International Standards for Tuberculosis Care *(ISTC): performance feedback and same-day smear microscopy. The strategies were selected based on a qualitative assessment guided by the Theory of Planned Behavior and the PRECEDE model. We collected patient data 6 months before and after the introduction of each intervention component, and compared ISTC adherence in the pre- and post-intervention periods for adults with cough ≥ 2 weeks’ duration.

**Results:**

The performance feedback evaluation included 1,446 adults; 838 (58%) were evaluated during the pre-intervention period and 608 (42%) during the post-intervention period. Performance feedback resulted in a 15% (95%CI +10% to +20%, p<0.001) increase in the proportion of patients receiving ISTC-adherent care. The same-day microscopy evaluation included 1,950 adults; 907 (47%) were evaluated during the pre-intervention period and 1,043 (53%) during the post-intervention period. Same-day microscopy was associated with a 14% (95%CI +10% to +18%, p<0.001) increase in the proportion of patients receiving ISTC-adherent care.

**Conclusions:**

Performance feedback and same-day microscopy should be considered along with ISTC training as part of a multi-faceted intervention to improve the quality of TB evaluation in other high TB burden countries.

## Introduction

Tuberculosis (TB) remains a major global public health crisis. Limiting transmission is essential to end the epidemic, and it is therefore important that cases be promptly diagnosed and started on treatment. However, at least 3 million of the estimated 8.6 million new cases in 2012 were not detected and reported to the World Health Organization (WHO) [1]. Under-diagnosis can be attributed to several factors, including poor access to health care, inadequate diagnostics, and poor quality of TB evaluation [2,3].

Widely accepted standards for TB evaluation have been described in the *International Standards for Tuberculosis Care* (ISTC) [4]. These include identifying patients with prolonged cough, referring patients with prolonged cough for sputum-based TB testing, and ensuring that patients with positive test results initiate treatment and are reported to public health authorities. In Uganda, we showed previously that adherence to these guidelines is poor [5]; similar findings have been reported in other high burden countries [5,6,7,8,9,10,11,12]. Improving implementation of TB evaluation guidelines is therefore critical for making progress towards TB elimination.

Health worker training has been the standard approach to guideline implementation. Although knowledge and capacity are important, increasing evidence shows that behavioral approaches that go beyond training are needed to facilitate sustained implementation of clinical practice guidelines. Successful implementation requires changing provider behavior, and theory-informed approaches are most likely to result in successful behavior change [13]. We therefore evaluated the feasibility and impact of components of a theory-informed, multi-faceted intervention on the quality of TB evaluation at health centers in Uganda.

## Materials and Methods

### Study setting

These studies took place at 6 government-run Level IV health centers in rural Uganda. These primary care centers provide general medical care and are staffed by one physician in-charge, 3–6 clinical officers, 2–4 nurses, and 1–2 pharmacists. In addition, each health center has a basic laboratory staffed by 1–3 laboratory technicians. The health centers are equipped to perform basic diagnostic testing, including sputum smear microscopy. The health centers typically serve approximately 1,000 patients per month, with one larger health center serving approximately 2,000 patients per month. Since 2009, our research team has monitored quality of TB evaluation at each health center using an electronic surveillance system that captures data on every clinical encounter. Data are abstracted from a paper case record form that consolidates demographic, clinical, laboratory, and pharmacy information recorded previously in multiple patient registers. The form has now been adopted by the Uganda Health Management Information System. A data entrant at each health center hired by the study cross-validated the data against separately maintained TB laboratory and treatment registers to ensure accuracy, and entered the data into and Epi Info (Centers for Disease Control and Prevention, Atlanta, GA, USA) database. Data are uploaded to a central server and used to assess standardized indicators based on the ISTC. Following implementation of the data collection system, research staff reviewed 1 year of data from TB laboratory and treatment registers to confirm accuracy of data entry.

### Theoretical model

**Fig 1:**
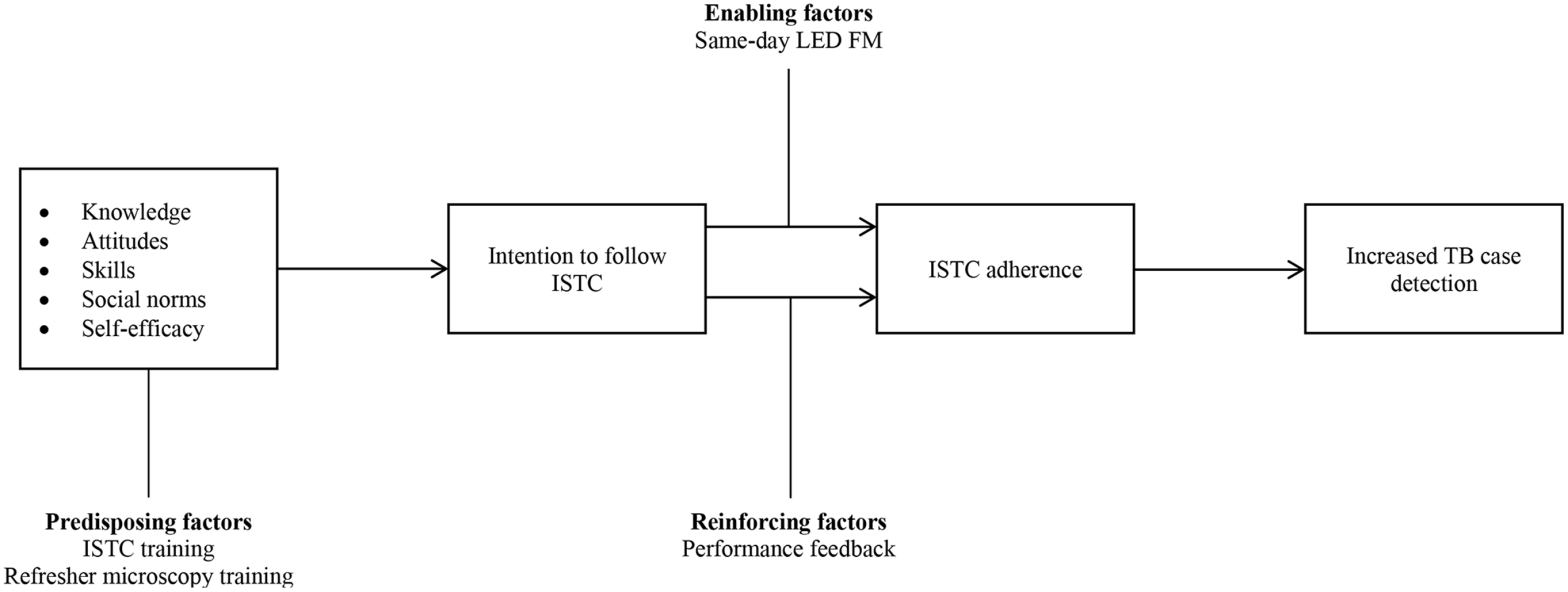
Overview of Intervention Development Process. To develop a multi-faceted intervention to improve adherence to the International Standards of TB Care (ISTC), we first developed a conceptual model based on the Theory of Planned Behavior. In this model, providers’ intention to follow ISTC-recommended TB evaluation practices is based on knowledge of and attitudes toward ISTC, having the required skills, perceived attitudes of their peers toward ISTC and belief in their ability to follow ISTC. Second, we conducted a formative assessment to gather data on these factors as well as health system barriers to TB evaluation. Finally, we selected intervention components that targeted pre-disposing, enabling, and reinforcing factors, as recommended by the PRECEDE model, and that were considered feasible and sustainable by key stakeholders.

We previously conducted a series of qualitative interviews in order to understand barriers to TB evaluation and inform intervention design [14]. We employed the Theory of Planned Behavior as the conceptual framework to guide this process (Fig 1). In a systematic review of guideline implementation studies, the Theory of Planned Behavior was the most likely theory to predict guideline adherence [15]. This theory asserts that intention is the best predictor of behavior and that three factors mediate the strength of intention: (1) attitudes (expected value of behavioral performance); (2) subjective norms (what important others think about the behavior); and (3) self-efficacy (perception of ability to overcome barriers to behavioral performance) [16].

Following barrier identification, we employed the PRECEDE model to select components of a multi-faceted intervention (Fig 1). We chose the PRECEDE model based on its strong empirical base and applicability to guideline adherence [17]. The model is based on three factors relevant to health behavior change: (1) *predisposing* factors—prior motives that either support or inhibit behavior; (2) *reinforcing* factors—rewards or punishments following a behavior or anticipated as a consequence of it; and (3) *enabling* factors—objective characteristics of an individual or environment that facilitate behavior [18]. A meta-analysis of 50 randomized controlled trials of continuing medical education demonstrated that the studies employing a combination of interventions representing PRECEDE categories were the most likely to alter physician behavior and influence patient outcomes [19]. Our intervention components targeted barriers identified in the formative assessment and grouped into the PRECEDE categories (Fig 1): (1) ISTC training to address pre-disposing factors and enhance behavioral capability; (2) performance feedback to reinforce ISTC adherence by facilitating observational learning and continuous quality improvement; and (3) single-specimen microscopy [20] to enable ISTC adherence by reducing the burden of diagnostic evaluation on patients and providers.

### Interventions

Because we previously conducted ISTC and refresher microscopy training as the first component of the intervention [5], this study focused on feasibility and impact of performance feedback and single-specimen microscopy. Performance feedback is a strategy employing regular monitoring and feedback to allow health care workers to critically analyze performance and identify areas for improvement. It has been shown to be effective for improving laboratory practices and quality of smear examination [21]. Our intervention involved delivery of a monthly Report Card, which displayed: (1) a health center’s performance on each ISTC indicator for the current month and for the previous 6 months and (2) performance data averaged across all six health centers. After staff introduced the Report Card at each health center, it was sent electronically each month to the health center in-charge or TB focal person. Health center staff were asked to review the Report Card at monthly staff meetings to devise a performance improvement plan. This continued monthly, with each new Report Card being used to evaluate the success of plans developed the previous month and determine the need for new actions. We hypothesized that observational learning and continuous quality improvement activities would reinforce adherence to ISTC-recommended TB evaluation practices by improving coordination and communication between health center staff and increasing the capacity of staff to identify and address problems.

The second intervention was same-day microscopy, which sought to enable ISTC adherence by reducing losses to follow-up during sputum smear evaluation. Up to 50% of patients do not return to receive results or initiate treatment with the standard, multi-day sputum collection and testing process [22,23,24,25,26,27]; in contrast, same-day microscopy involves collection and analysis of two sputum samples at a patient’s initial visit to the health center, facilitating same-day diagnosis and treatment initiation for smear-positive TB cases. We partnered with the Uganda National TB Reference Laboratory (NTRL) to conduct a 5-day, on-site training at each health center to replace conventional smear microscopy with same-day light emitting diode fluorescence microscopy (LED FM), which requires less technician time and reduces laboratory workload [28]. We trained staff in the proper use of LED fluorescence microscopes (Primo Star iLED, Carl Zeiss Microscopy, Germany) following manufacturer guidelines, and smear preparation and staining according to NTRL guidelines. Health center staff practiced preparing and staining smears using patient sputum samples, and interpreted both patient smears and known positive and negative controls using semi-quantitative scoring. We conducted proficiency testing using panel slides prepared at the NTRL, and required a mark of ≥80% to pass. A lab officer performed monthly external quality assurance testing using standard lot quality assurance sampling for three months following implementation; this was followed by routine quarterly external quality assurance performed by the NTRL [29]. We hypothesized that same-day LED FM would enable adherence to the ISTC by improving the capacity of providers to diagnose TB and start treatment during a patient’s initial visit to the health center.

### Evaluation

We evaluated the feasibility and impact of each intervention component separately using a quasi-experimental design. The performance feedback evaluation was conducted from February 2010 through June 2011 and the same-day microscopy evaluation from August 2011 through March 2012. For each evaluation, study staff introduced the intervention component at each of the 6 health centers in a randomly determined order (S1 Fig). We collected patient data including demographics, cough history, sputum smear referrals and results, and TB treatment prescriptions 6 months before and after the introduction of the intervention at each health center. We included data on all adults reporting cough ≥2 weeks’ duration, and excluded data on patients for whom cough history was not known and on patients returning for TB medication refills.

### Outcomes

The primary outcome was the proportion of adults with cough ≥2 weeks receiving ISTC-adherent care. Secondary outcomes included each component of ISTC-adherent care: the proportion of adults with cough ≥2 weeks referred for sputum examination, the proportion of adults with cough ≥2 weeks completing sputum examination if referred, and the proportion of smear-positive TB cases initiating TB treatment.

### Analysis

We assessed differences in patient characteristics and outcomes between the pre- and post-intervention periods using the chi-squared or Fisher’s exact tests. We developed logistic regression models to estimate the effect of the intervention on each outcome, adjusting for age, sex, and intervention site. We generated piecewise linear regression models to test for underlying secular trends within the pre- and post-intervention periods. Based on data from 2009, we estimated that at least 1,450 patients would be included across the six health centers (725 each in the pre- and post-intervention periods). With this sample, we estimated we would have 90% power to detect absolute increases in the proportions receiving the components of ISTC-adherent care of approximately 3–9%, depending on the proportion in the pre-intervention period.

### Ethics approval

The University of California San Francisco Committee on Human Research, Makerere University School of Medicine Research Ethics Committee, and Uganda National Council of Science and Technology approved this study and waived the requirement for informed consent. No patient identifiable information was collected.

## Results

### Performance feedback

#### Study population

Of 121,527 adults evaluated at the 6 health centers during the 12-month performance feedback evaluation, 116,195 (96%) did not have cough of at least 2 weeks’ duration, 2,957 (2.4%) did not have cough history recorded, and 302 (0.2%) presented for TB medication refills. Of the remaining 1,446 adults with cough of at least 2 weeks’ duration, 838 (58%) were evaluated during the pre-intervention period and 608 (42%) during the post-intervention period (S2 Fig). Median age (38 years vs. 38 years, p = 0.72) and the proportion female (54% vs. 50%, p = 0.17) were similar in the pre- and post-intervention periods.

#### Implementation

The performance feedback intervention had good uptake at 4 of 6 sites, as determined by staff reporting that the Report Card was discussed at monthly staff meetings. Examples of problems discussed and solutions identified included: (1) the clinic in-charge providing reminders to address clinicians forgetting to order TB exams; (2) the TB focal person providing training on TB evaluation guidelines to all new staff to address high turnover; and (3) establishing systems to borrow supplies and drugs from neighboring clinics to address inconsistent delivery from supply chains. The two sites with poor uptake did not hold routine monthly staff meetings. However, the Report Cards were reviewed at quarterly meetings led by research staff.

#### Outcomes

In bivariate analysis, patients in the post-intervention period were more likely to receive ISTC-adherent care compared with patients in the pre-intervention period (67% vs. 52%, difference +16%, 95%CI +11% to +21%, p<0.001, Table 1). Patients in the post-intervention period were more likely to be referred for sputum examination (82% vs. 72%, difference +10%, 95%CI +6% to +15%, p<0.001), complete sputum examination following referral (84% vs. 74%, difference +10%, 95%CI +5% to +15%, p<0.001), and initiate treatment if smear-positive (86% vs. 72%, difference +13%, 95%CI +1% to +28%, p = 0.07), though the latter result was not statistically significant. In addition, the proportion of patients undergoing testing who were found to be sputum smear-positive was higher in the post-intervention period (11% vs. 7%, difference +5%, 95%CI +1% to +8%, p = 0.01).

**Table 1:** Bivariate analysis of intervention impact on presumed TB patient evaluation at 6 health centers.

	Pre-intervention	Post-intervention	Difference	p-value
**Performance feedback**	**N = 838**	**N = 608**		
Received ISTC-adherent care	432 (52%)	410 (67%)	+16% (+11% to +21%)	<0.001
Referred for sputum examination	601 (72%)	499 (82%)	+10% (+6% to +15%)	<0.001
Completed sputum examination	447 (74%)	420 (84%)	+10% (+5% to +15%)	<0.001
Initiated treatment if sputum smear-positive	39 (72%)	59 (86%)	+13% (+1% to +28%)	0.07
**Same-day LED FM**	**N = 907**	**N = 1,043**		
Received ISTC-adherent care	527 (58%)	784 (75%)	+17% (+13% to +21%)	<0.001
Referred for sputum examination	706 (78%)	816 (78%)	+0.4% (-3% to +4%)	0.83
Completed sputum examination	531 (75%)	786 (96%)	+21% (+18% to +25%)	<0.001
Initiated treatment if sputum smear-positive	87 (96%)	77 (97%)	+2% (-4% to +7%)	0.69

After adjusting for age, sex, and site, there was an absolute 15% (95%CI 10% to 20%, p<0.001) increase in the proportion of patients receiving ISTC-adherent care in the post-intervention period (Table 2). The proportion of patients referred for sputum examination (difference +9%, 95%CI +5% to +14%, p<0.001) and completing sputum examination following referral (difference +9%, 95%CI +4% to +13%, p<0.001) were higher in the post-intervention period (Table 2). In addition, the proportion of patients initiating treatment if sputum-smear positive (difference +12%, 95%CI -4% to +27%, p = 0.14) was higher in the post-intervention period, but the result was not statistically significant. There were no secular trends in the pre- or post-intervention periods for the primary outcome or secondary outcomes (S3 Fig). The prevalence of smear-positive TB among patients with cough of at least 2 weeks’ duration increased from 64 per 1000 in the pre-intervention period to 113 per 1000 in the post-intervention period (difference +50 per 1000, 95% CI +20 per 1000 to +80 per 1000).

**Table 2:** Multivariate analysis of intervention impact on presumed TB patient evaluation at 6 health centers.

	Adjusted Proportion^a^			
	Pre-intervention	Post-intervention	Difference	p-value
**Performance feedback**	**N = 838**	**N = 608**		
Received ISTC-adherent care	52% (49%-55%)	67% (63%-70%)	+15% (+10% to +20%)	<0.001
Referred for sputum examination	72% (69%-75%)	82% (79%-85%)	+9% (+5% to +14%)	<0.001
Completed sputum examination	75% (72%-78%)	84% (80%-87%)	+9% (+4% to +13%)	<0.001
Initiated treatment if sputum smear-positive	69% (57%-81%)	81% (72%-91%)	+12% (-4% to +27%)	0.14
**Same-day LED FM**	**N = 907**	**N = 1,043**		
Received ISTC-adherent care	60% (57%-63%)	74% (72%-7%)	+14% (+10% to +18%)	<0.001
Referred for sputum examination	77% (77%-82%)	78% (75%-80%)	-1% (-5% to +2%)	0.43
Completed sputum examination	77% (74%-79%)	96% (95%-97%)	+19% (+16% to +23%)	<0.001
Initiated treatment if sputum smear-positive	91% (82%-99%)	92% (82%-100%)	+2% (-12% to +15%)	0.82

^a^Adjusted for age, sex, and site.

### Same-day LED Fluorescence Microscopy

#### Study population

Of 89,474 adults evaluated at the 6 health centers during the 12-month evaluation of same-day LED FM, 87,412 (98%) did not have cough of at least 2 weeks’ duration, 68 (<1%) did not have cough history recorded, and 44 (<1%) presented for TB medication refills. Of the remaining 1,950 adults with cough of at least 2 weeks’ duration, 907 (47%) were evaluated during the pre-intervention period and 1,043 (53%) during the post-intervention period (S4 Fig). Median age (37 years vs. 38 years, p = 0.11) and the proportion female (46% vs. 50%, p = 0.14) were similar in the pre- and post-intervention periods.

#### Implementation

There was good uptake of same-day LED FM at all six sites; all technicians passed proficiency testing immediately post-training, and no major quantification errors were observed during the external quality assurance testing. In addition, to facilitate same-day reporting of results, all sites implemented a cough register to facilitate early referral of patients to the laboratory for sputum collection and switched from batched to on-demand preparation and analysis of sputum smears.

#### Outcomes

In bivariate analysis, patients in the post-intervention period were more likely to receive ISTC-adherent care compared with patients in the pre-intervention period (75% vs. 58%, difference +17%, 95%CI +13% to +21%, p<0.001, Table 1). There was no difference in the proportion of patients with cough ≥2 weeks’ duration referred for sputum examination (78% vs. 78%, difference +0.4%, 95%CI -3% to +4%, p = 0.83). However, patients in the post-intervention period were more likely to complete sputum examination if referred (96% vs. 75%, difference +21%, 95%CI +18% to +25%, p<0.001). The proportion of patients initiating treatment if sputum smear-positive was high in the pre- and post-intervention periods (96% vs. 97% post-intervention, difference +2%, 95%CI -4% to +7%, p = 0.69). The proportion of patients found to be sputum smear-positive was similar in the post-intervention period compared with the pre-intervention period (8% vs. 10%, difference -2%, 95%CI -5% to +0.1%, p = 0.06).

There was a downward secular trend in the proportion of patients receiving ISTC-adherent care in the pre-intervention period (S5 Fig). After adjusting for age, sex, and site, there was a 14% (95%CI +10% to +18%, p<0.001) absolute increase in the proportion of patients receiving ISTC-adherent care in the post-intervention period (Table 2). The proportion of patients referred for sputum examination did not change in the post-intervention period (difference -1%, 95%CI -5% to +2%, p = 0.43), however there was a 19% (95%CI +16% to +23%, p<0.001) increase in the proportion of patients completing sputum examination if referred. There was no change in the proportion initiating treatment if sputum AFB smear-positive in the post-intervention period (difference +2%, 95%CI -12% to +15%, p = 0.82). The prevalence of smear-positive TB among patients with cough of at least 2 weeks’ duration was similar in the pre- and post-intervention periods (100 per 1000 vs. 80 per 1000, difference -20 per 1000, 95%CI -40 per 1000 to +70 per 1000).

### Between-site variability

Performance feedback results were fairly consistent across sites, with 4 of 6 showing significant improvement in the proportion of patients receiving ISTC-adherent care in the post-intervention period (range 12%-30%, Table 3). One site (Health Center B) did not show improvement despite implementing the intervention. There was no improvement at one site with inconsistent uptake of the intervention (Health Center F, +1%, 95%CI -15% to +17%, p = 0.15), however the other site with inconsistent uptake showed improvement (Health Center E, +12%, 95%CI +1% to +23%, p = 0.04).

**Table 3:** Between-site variation in impact of interventions on ISTC-adherent care at 6 health centers.

	Adjusted Proportion^a^ (95% CI)			
	Pre-intervention	Post-intervention	Difference	p-value
**Performance feedback**	**N = 838**	**N = 608**		
Health Center A	57% (49%-65%)	88% (82%-94%)	+30% (+20% to +40%)	<0.001
Health Center B	73% (63%-83%)	74% (62%-86%)	+1% (-15% to +17%)	0.90
Health Center C	75% (68%-82%)	87% (81%-93%)	+12% (+2% to +21%)	0.01
Health Center D	49% (42%-56%)	64% (56%-72%)	+15% (+5% to +26%)	0.01
Health Center E	30% (23%-37%)	42% (33%-50%)	+12% (+1% to +23%)	0.04
Health Center F	32% (23%-40%)	43% (30%-56%)	+12% (-4% to +27%)	0.15
**Same-day LED FM**	**N = 907**	**N = 1,043**		
Health Center A	84% (78%-90%)	82% (76%-87%)	-2% (-11% to +6%)	0.55
Health Center B	93% (88%-98%)	94% (89%-98%)	+1% (-6% to +8%)	0.85
Health Center C	63% (56%-70%)	74% (68%-81%)	+11% (+1% to +21%)	0.03
Health Center D	81% (74%-87%)	79% (73%-85%)	-2% (-11% to +7%)	0.65
Health Center E	43% (36%-50%)	67% (61%-74%)	+25% (+15% to +34%)	<0.001
Health Center F	17% (12%-22%)	60% (53%-67%)	+43% (+34% to +52%)	<0.001

^a^Adjusted for age and sex

With same-day LED FM, a higher proportion of patients received ISTC-adherent care in the post-intervention period at three of six sites (Table 3). In addition, 4 of 6 sites showed substantial improvement in the proportion of patients completing smear examination (S1 Table). The other 2 sites also showed improvement, though the results were not statistically significant. The proportion of smear-positive patients who initiated treatment was 100% at 5 of 6 sites in the post-intervention period (S2 Table).

## Discussion

In this study, we showed that performance feedback and same-day microscopy each improved the quality of TB evaluation at health centers in Uganda. These relatively low-cost interventions are complementary and should be evaluated together in other settings with similar barriers to TB evaluation. Such health system interventions are critical for maximizing the impact of novel diagnostics on case detection and treatment.

Performance feedback, also referred to as audit and feedback, has been widely studied, but has had varied results. In a systematic review of 140 randomized controlled trials, Ivers et al found that audit and feedback interventions led to a median adjusted increase of 4.3% in the desired behavior, but individual study estimates ranged from a 9% decrease to a 70% increase [30]. The authors identified 5 factors associated with greater impact: low baseline performance, feedback coming from a supervisor or colleague, feedback provided multiple times, feedback delivered in both verbal and written form, and feedback including explicit targets and an action plan. Notably, all five factors were present in our study: baseline adherence to ISTC-recommended TB evaluation practices was poor; feedback was provided on a monthly basis by the local TB focal person or clinic in-charge; and feedback included both a written report card with explicit targets and verbal discussion of the report card to develop an action plan. We also designed our performance feedback intervention based on well-established theoretical constructs [31], which is known to improve the success of complex interventions [13]. Behavior change theories indicate that feedback can make providers aware that current practices are inconsistent with those of peers or guidelines, and can change beliefs about the consequences of current practices, social norms, and perceived ability to perform the desired behavior. Thus, audit and feedback complements training, which improves knowledge and skills and other pre-disposing factors that affect intention to follow guidelines.

Similarly, same-day LED FM complements both ISTC training and performance feedback by addressing recurring problems related to self-efficacy, including the high workload of laboratory staff and failure of patients to return after the initial visit to complete TB evaluation. These factors are beyond the control of providers and contribute to sub-standard TB evaluation even when providers have the appropriate knowledge and skills and strong intentions to follow guidelines. Following implementation of same-day LED FM, the proportion of patients completing smear examination increased from 77% to 96%, and overall 97% of smear-positive patients initiated treatment. This demonstrates that sputum smear examination and initial clinical management, including treatment initiation for those that need it, can be consistently completed at patients’ initial clinic visit. Importantly, same-day diagnosis and treatment of smear-positive TB occurred without the need for additional human resources. Same-day microscopy also benefits smear-negative patients who either do not need to make a return clinic visit if feeling better or could be assessed more promptly for other potential illnesses.

Our study has a number of strengths. First, we provide evidence supporting the use of interventions based on theoretical frameworks to promote provider behavior change. We identified interventions targeting factors that both *reinforced* and *enabled* adherence to standards for TB care [14]. Second, while many studies evaluating behavior change make conclusions based on self-report [32], our outcome measures were objective. Third, our study focused on the TB evaluation process as a whole rather than on isolated pieces. For performance feedback, we provided health care workers with information about their performance on all ISTC indicators, allowing them to tailor their responses. Furthermore, monitoring was ongoing, making it possible for health workers to see the impact of their solutions over time. Likewise, the same-day microscopy intervention targeted two crucial areas of the diagnostic pathway: testing completion and treatment initiation. Many studies concentrate on improving a single element of the diagnostic pathway, such as laboratory performance or reporting of smear results [21,33,34,35,36,37]. While these strategies may have an impact, approaches that influence multiple barriers are likely more effective [30]. Finally, we worked in health centers typical of rural Africa, indicating that our findings may be generalizable to other high-burden, low-resource settings.

Our study has several limitations. First, pre-post study designs can inaccurately attribute observed improvements to the intervention under evaluation. To address this, we collected outcome data at multiple time points and checked for underlying secular trends across both study periods. We observed a downward secular trend occurring prior to the same-day microscopy intervention, suggesting that we may have in fact underestimated the impact of the intervention. Second, implementation of the performance feedback intervention was variable. Of the two sites that did not improve, one was performing moderately well in the pre-intervention period and the other did not implement the program according to the protocol because the health center in-charge was not supportive of quality improvement initiatives. These findings support that performance feedback is effective, and should be targeted to poorly performing sites. But to maximize effectiveness, organizations and implementation should consider health center readiness for change when implementing quality improvement interventions. In addition, it is notable that ISTC-adherence declined at some sites in the period following the performance feedback intervention and prior to implementation of same-day microscopy. This indicates that performance feedback should either be ongoing or implemented for more than six months before being discontinued in order to achieve lasting improvements. Finally, the study design cannot rule out that the success of intervention components introduced at a later time point depended on the introduction and success of components introduced earlier. However, the components were conceived to be part of a multi-faceted intervention and we demonstrate that each component provided additional value beyond what was done previously.

In conclusion, the quality of TB care at routine health centers must be improved in order to achieve the WHO post-2015 development framework goal of ending the global TB epidemic by 2035 [1]. We found that performance feedback and same-day microscopy–two interventions selected based on a theory-informed assessment of behavioral and environmental barriers to TB evaluation–measurably improved the quality of TB evaluation at rural health centers in Uganda. Further studies are needed to evaluate the impact of a multi-faceted intervention including ISTC training, performance feedback and same-day microscopy on the quality of TB care in other settings and to quantify its impact on TB case detection and treatment.

## Supporting Information

S1 Dataset Performance feedback dataset. (XLS)

S2 Dataset Same-day LED FM dataset. (XLS)

S1 Fig Schedule for introducing interventions at 6 health centers. Performance feedback evaluation conducted February 2010–June 2011; same-day microscopy evaluation conducted August 2011–March 2012.
(TIF)

S2 Fig Performance feedback enrollment. (TIF)

S3 Fig Adjusted time trend for receiving ISTC-adherent care at 6 health centers in pre- and post-intervention periods: Performance feedback. Trend pre-intervention: p = 0.78; trend post-intervention: 0 = 0.67.
(TIF)

S4 Fig Same-day LED FM enrollment. (TIF)

S5 Fig Adjusted time trend for receiving ISTC-adherent care at 6 health centers in pre- and post-intervention periods: Same-day microscopy. Trend pre-intervention: p<0.001; trend post-intervention: p = 0.40.
(TIF)

S1 Table Impact of same-day LED FM on completing sputum examination at 6 health centers. (DOCX)

S2 Table Impact of same-day LED FM on initiating treatment if sputum smear-positive at 6 health centers. (DOCX)
