# Supplementary figures and images for: Theory-Informed Interventions to Improve the Quality of Tuberculosis Evaluation at Ugandan Health Centers: A Quasi-Experimental Study
Source: PLoS One. 2015 Jul 14;10(7):e0132573. doi: 10.1371/journal.pone.0132573 (PMC4501843; doi:10.1371/journal.pone.0132573)

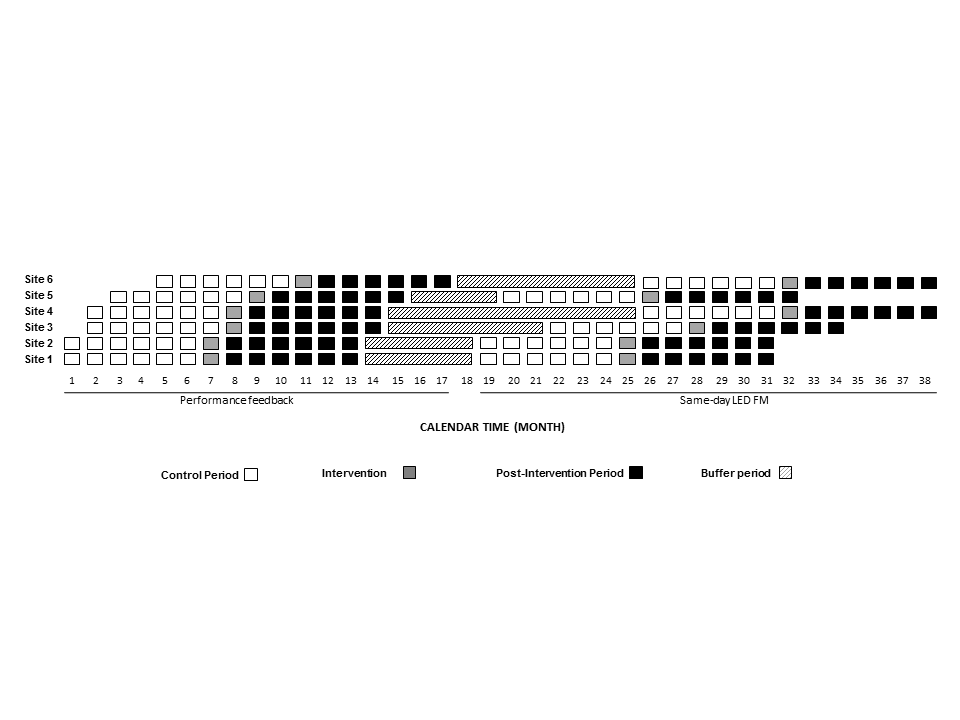

Supplement: S1 Fig — Performance feedback evaluation conducted February 2010–June 2011; same-day microscopy evaluation conducted August 2011–March 2012. (TIF) [file pone.0132573.s003.tif]

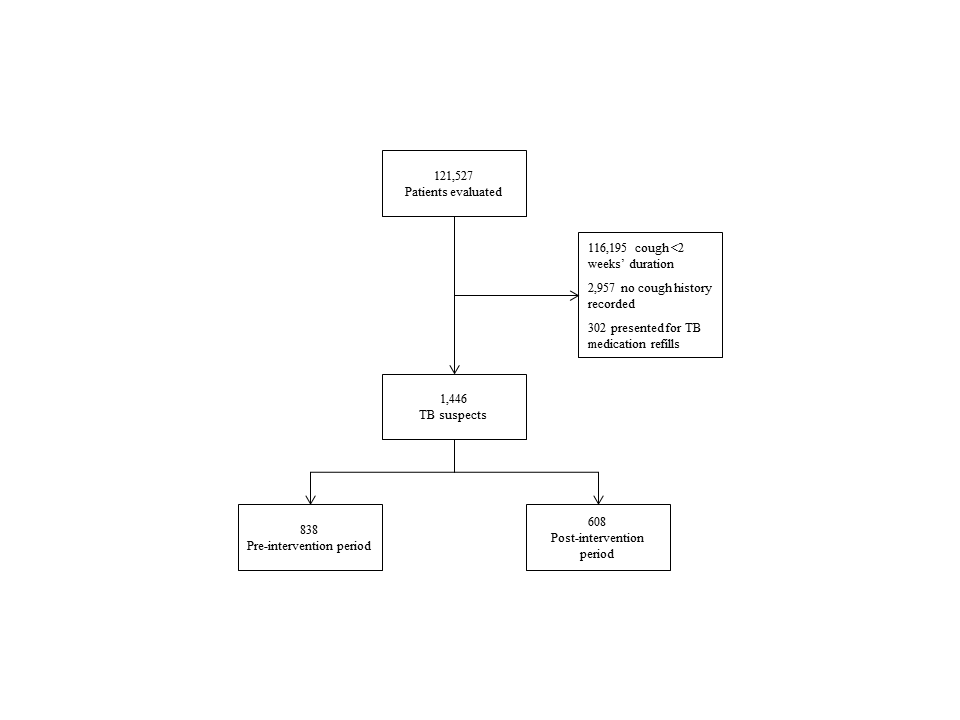

Supplement: S2 Fig — (TIF) [file pone.0132573.s004.tif]

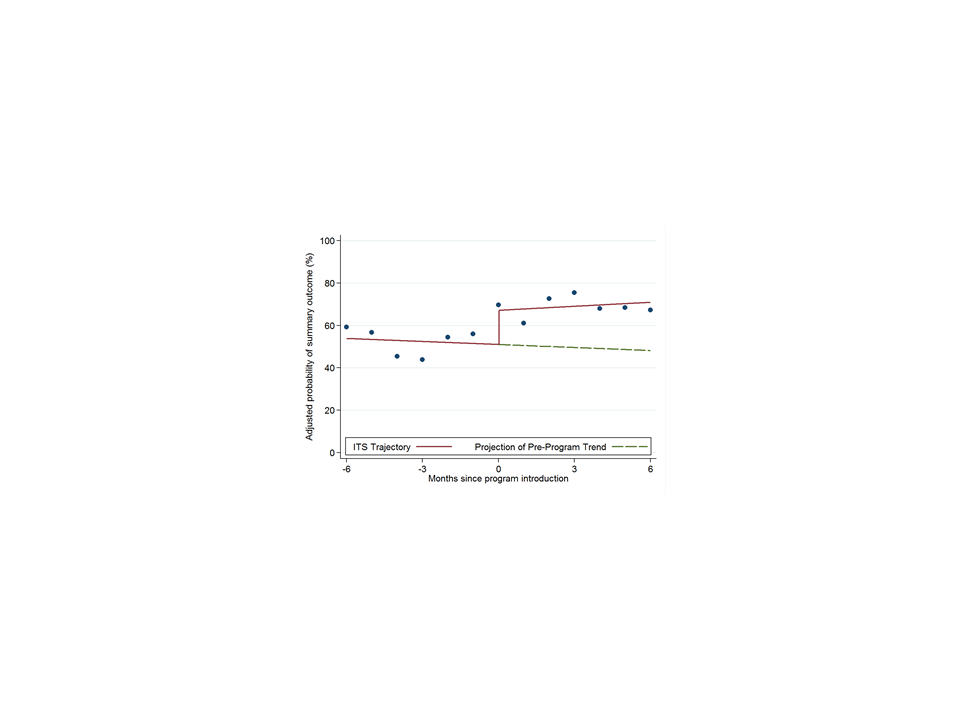

Supplement: S3 Fig — Trend pre-intervention: p = 0.78; trend post-intervention: 0 = 0.67. (TIF) [file pone.0132573.s005.tif]

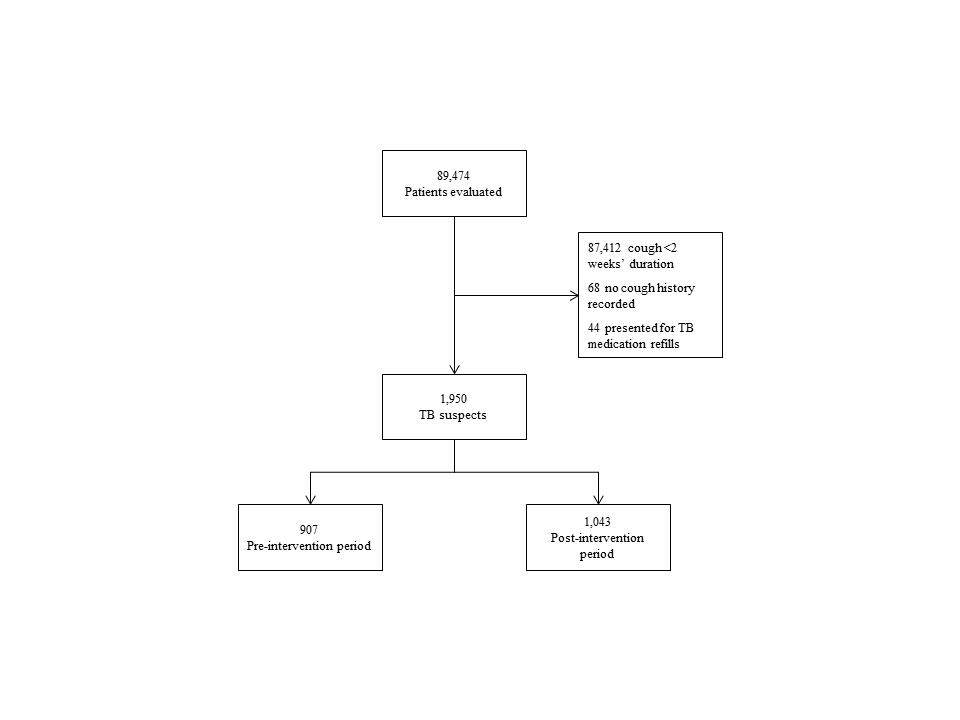

Supplement: S4 Fig — (TIF) [file pone.0132573.s006.tif]

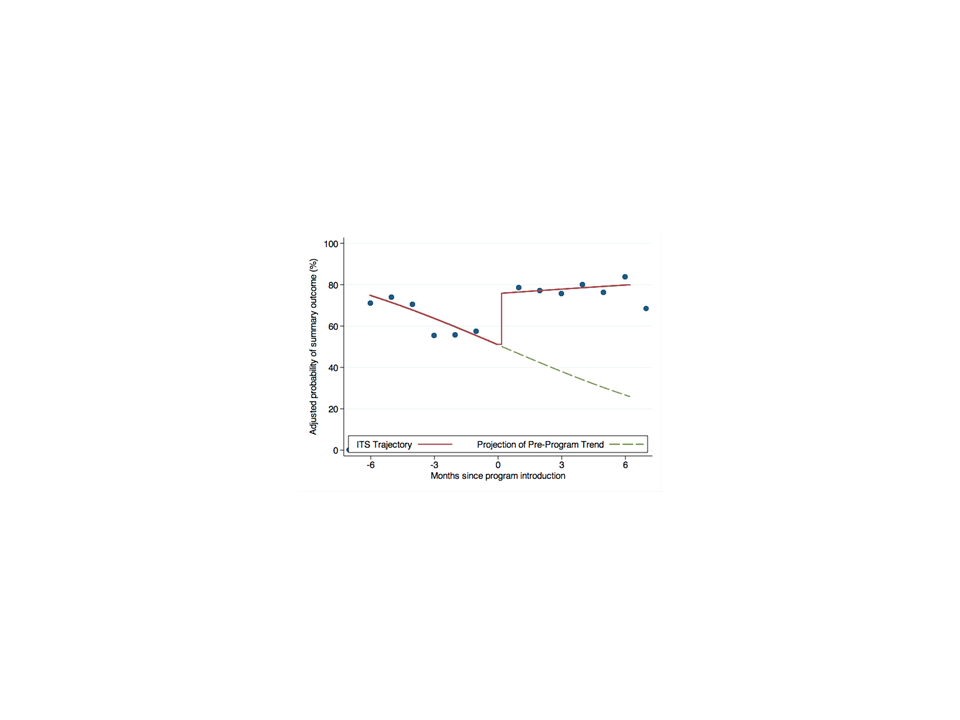

Supplement: S5 Fig — Trend pre-intervention: p<0.001; trend post-intervention: p = 0.40. (TIF) [file pone.0132573.s007.tif]
