# Supplementary material for: Theory-Informed Interventions to Improve the Quality of Tuberculosis Evaluation at Ugandan Health Centers: A Quasi-Experimental Study
Source: PLoS One. 2015 Jul 14;10(7):e0132573. doi: 10.1371/journal.pone.0132573 (PMC4501843; doi:10.1371/journal.pone.0132573)
Supplement: S1 Table — (DOCX) [file pone.0132573.s008.docx]

|  | **Adjusted Proportion^a^ (95% CI)** | |  |  |
| --- | --- | --- | --- | --- |
|  | **Pre-intervention** | **Post-intervention** | **Difference** | **p-value** |
| Health Center A | 91% (86%-96%) | 95% (91%-98%) | +4% (-2% to +10%) | 0.24 |
| Health Center B | 97% (93%-100%) | 98% (96%-100%) | +1% (-3% to +6%) | 0.60 |
| Health Center C | 75% (68%-82%) | 92% (8%7-97%) | +17% (+8% to +25%) | <0.001 |
| Health Center D | 88% (82%-94%) | 99% (98%-100%) | +11% (+6% to +17%) | <0.001 |
| Health Center E | 70% (62%-79%) | 99% (98%-100%) | +29% (+20% to +37%) | <0.001 |
| Health Center F | 30% (22%-39%) | 94% (90%-99%) | +64% (+54% to +74%) | <0.001 |
| ^a^Adjusted for age and sex |  |  |  |  |
